# Reference values of impulse oscillometry (IOS) for healthy Chinese children aged 4–17 years

**DOI:** 10.1186/s12931-022-02080-z

**Published:** 2022-07-12

**Authors:** Jinhong Wu, Hao Zhang, Yongsheng Shi, Jinrong Wang, Yuling Han, Qiaoling Zhang, Ning Wang, Sha Liu, Yuehua Zhang, Huifen Zi, Fei Wang, Aihong Liu, Yuxin Song, ChunMei Jia, Yong Feng, Quanhua Liu, liya Wan, Minghong Ji, Zhen Long, Jianfeng Huang, Li Liu, Yun Sun, Suping Tang, Xiaoyan Dong, Xiaojian Zhou, Wenhui Jiang, Li Shen, Haohua Jiang

**Affiliations:** 1 Department of Respiratory Medicine, Shanghai Children’s Medical Center, School of Medicine, Shanghai Jiao Tong University, Shanghai, 200127 China; 2 Department of Internal Medicine, Shanghai Children’s Medical Center, School of Medicine, Shanghai Jiao Tong University, Shanghai, 200127 China; 3 Department of Pediatric Respiratory, Maternity and Child-Care Hospital of Gansu Province, Lanzhou, 730050 China; 4 Department of Pediatric Respiratory, Affiliated Provincial Hospital of Shandong First Medical University, Jinan, 250021 China; 5 Department of Respiratory, Qilu Children’s Hospital of Shandong University, Jinan, 250022 China; 6 Department of Pediatric Respiratory, Maternal and Child Health Hospital in Inner Mongolia Autonomous Region, Hohhot, 010020 China; 7 Asthma Centre of Xi’an Children’s Hospital, Xi’an, 710003 China; 8 Department of Respiratory Medicine, Children’s Hospital of Chongqing Medical University, Chongqing, 400014 China; 9 Pediatrics Infection Disease Ward, Central South University Xiangya School of Medicine Affiliated Haikou Hospital, Haikou, 570208 China; 10 Department of Pediatrics, Baotou Central Hospital, Baotou, 014040 China; 11 Department of Pediatric Respiratory, Guiyang Maternal and Child Health Hospital (Guiyang Children’s Hospital), Guiyang, 550003 China; 12 Department of Respiratory Medicine, Children’s Hospital of Shanxi, Taiyuan, 030013 China; 13 Department of Allergy, Harbin Children’s Hospital, Harbin, 150010 China; 14 Department of Pediatric Respiratory, The Fourth Hospital of Baotou (Baotou Children’s Hospital), Baotou, 014030 China; 15 Department of Pediatrics, Shengjing Hospital of China Medical University, Shenyang, 110004 China; 16 Department of Pulmonology, Xinhua Hospital Affiliated to Shanghai Jiao Tong University School of Medicine, Shanghai, 200092 China; 17 Respiratory department of Tianjin Children’s Hospital, Tianjin, 300074 China; 18 Department of Pediatric, The First Affiliated Hospital of USTC Anhui Provincial Hospital, Hefei, 230001 China; 19 Department of Pediatric Respiratory Medicine, Maternal and Child Health Hospital of Hubei Province, Tongji Medical College, Huazhong University of Science and Technology, Wuhan, 430070 China; 20 Department of Pulmonology, Children’s Hospital of Fudan University, Shanghai, 201102 China; 21 Department of Pediatric Respiratory, The First Hospital of Jilin University, Changchun, 130021 China; 22 General Pediatric, Yinchuan Women and Children Healthcare Hospital, Yinchuan, 750001 China; 23 Department of Asthma and Tracheitis, Fuzhou Children’s Hospital, Fujian Medical University, Fuzhou, 350000 China; 24 Department of Pulmonology, Shanghai Children’s Hospital, Affiliated to Shanghai Jiao Tong University, Shanghai, 200040 China; 25 Department of Pediatrics, Shanghai First People’s Hospital, Affiliated to Shanghai Jiao Tong University, Shanghai, 200080 China; 26 Department of Respiratory, Guangzhou Women and Children’s Medical Center, Guangzhou, 510623 China; 27 Department of Respiratory, Shanghai Chest Hospital Affiliated to Shanghai Jiaotong University, Shanghai, 200025 China

**Keywords:** Impulse oscillometry, Pulmonary function test, Normal predicted value, Child

## Abstract

**Objective:**

To establish the predicted value of pulmonary function determined by impulse oscillometry (IOS) in children (4–17 years old) in China.

**Methods:**

A total of 6270 healthy children aged 4–17 years in China were included. The Master Screen IOS pulmonary function device (Jaeger Co, Germany) was used to detect the respiratory impedance (Zrs), resonant frequency (Fres), respiratory system resistance (Rrs) and respiratory system reactance (Xrs) at various oscillation frequencies, and the indices above were analysed. Stepwise multivariate regression was used to establish the regression equation of related parameters of IOS in different sexes, ages, height, and weight.

**Results:**

The differences in the main IOS parameters between different age stages were statistically significant regardless of sex (*P* < 0.05). The stepwise multivariate regression analysis showed that IOS parameters were related to height, age, and weight, and most IOS parameters were most closely related to height (the absolute value of the regression coefficient was the largest). With increasing age and height, the values of Z_5_, R_5_, R_20_, R_5_–R_20_, and Fres decreased, while the value of X_5_ increased. Through height, age, and weight, we obtained the normal predicted values equation of children’s IOS parameters. Compared with the other reference equations, our reference equation is more suitable for Chinese children.

**Conclusions:**

The study revealed the reference values of IOS parameters in healthy Chinese children. In the evaluation of results for lung function measurements, this predicted value equation is more consistent with the characteristics of Chinese children than other reference equations.

*Clinical Trial*: ChiCTR: 1800019029.

**Supplementary Information:**

The online version contains supplementary material available at 10.1186/s12931-022-02080-z.

## Introduction

Chronic respiratory diseases, such as asthma, have an increasing incidence among children in developing countries, and both the morbidity and mortality of respiratory diseases rank first among all systemic diseases in paediatrics [1]. Lung function is an important aspect of respiratory physiology and a major indicator of early diagnosis and control of respiratory diseases [2]. The commonly proposed method to assess lung function to date is spirometry, which requires active participation, leading to the challenge of achieving a reproducible and reliable measurement in children. The recent American Thoracic Society (ATS)/European Respiratory Society (ERS) joint statement on lung function testing in preschoolers summarized the use of different techniques in children and highlighted the importance of forced oscillation techniques (impulse oscillometry system (IOS)) in children [3]. In the principle of forced oscillation, a rectangular electric pulse is generated by a pulse generator and superimposed on the resting breath of the subject. Through Fourier transform and spectrum analysis, different respiratory impedance values (respiratory system resistance, respiratory system reactance, and inertial resistance) at different frequencies can be calculated. The impulse oscillation method (IOS) can detect airway resistance and calculate compliance by using sound waves with frequencies from 5 to 35 Hz. IOS is a simple and noninvasive technique for respiratory function testing during tidal breathing. It does not require the patient's special cooperation and is simple, noninvasive, repeatable, and provides comprehensive respiratory physiological parameters. It is especially suitable for people aged 3 and above [4, 5].

Establishing appropriate reference values can effectively help clinicians evaluate lung function and make accurate diagnoses and treatments. The ATS and ERS suggest that the establishment of reference values of lung function cannot be asserted in general terms but should consider factors such as race, living environment and age [6, 7]. Although some studies have focused on developing new reference equations, with specific dependencies mainly on children's height, weight and sex, the available reference formulas are mostly extrapolated from white children of European origin, and the results remain limited and inconsistent [8–14]. Moreover, China has followed foreign standards. In Taiwan, only one study of 150 children under 6 years of age attempted to establish this standard reference equation for Chinese children and found that height was the most important determinant of airway resistance [15]. However, there is no evidence showing that reference equations are suitable for school-aged children and teenagers. The lack of reference equations and normal IOS ranges for Chinese children seriously affect the accuracy of assessment and hinder the clinical application of the oscillation method.

Therefore, Shanghai Children's Medical Center was taken as the lead unit, and the paediatric respiratory departments of twenty-four specialized or general hospitals from 17 provinces in China joined to conduct the measurement of IOS in 6270 healthy children from May 2017 to May 2019, aiming to establish reference equations of IOS parameters that are based on data of healthy Chinese children collected from a wide region under standardized quality control to provide the basis for the rational clinical application of IOS.

## Materials and methods

### Participants

This is a cross-sectional study of kindergarten children and school-age students aged 4–17 in China from May 2017 to May 2019. Informed consent was obtained from the parents or legal guardians of the children. The exclusion criteria were as follows: ① respiratory tract infection occurring 4 weeks before the investigation; ② history of prematurity, congenital heart disease or respiratory, neuromuscular, immune and other diseases; ③ family history of asthma, rhinitis or other genetic allergic diseases; ④ inability to cooperate with the completion of pulse oscillation lung function detection; ⑤ abnormal pulmonary ventilation function; and ⑥ living in a family where people smoke. A total of 7021 children were enrolled in the study. This study was approved by the Ethics Committee of Shanghai Children's Medical Center (Ethics No: SCMCIRB-K2017007), and each subcentre follows the master research unit ethics. Clinical Trial: ChiCTR: 1800019029.

### Procedures

#### Physical growth index measurement

The height and body weight of all the subjects were measured by fixed personnel. The subjects wore shirts or thin sweaters (air-conditioned rooms) and took off their shoes. Their height was measured by a vertical altimeter, accurate to 0.1 cm, and their weight was measured by an electronic scale, accurate to 0.1 kg. The gender and birth dates of the children were recorded at the same time.

#### Pulmonary function test

All children were tested for pulmonary function on the day of the physical examination. IOS, then PFT, if abnormal spirometry, IOS data will not be used. The Masterscreen IOS system from Jaeger, Germany, is adopted and operated by specially trained PFT technicians.

IOS manoeuvres were performed in the sitting position. All operations and procedures were in accordance with ATS (American Thoracic Society, ATS), the ERS (European Respiratory Society, ERS) and the Chinese children's pulmonary function testing guidelines [6, 7, 17–19]. We used a bacterial filter, and impedance values were corrected for the impedance of the filter. The flow sensor should be calibrated once every day when the machine is started up and once again after replacing the flow sensor to ensure the accuracy of the flow and volume tests. The machine should be warmed up for 20 min after starting up every day, and then it should be calibrated. Prior to the measurement, the investigator should perform a quick visual check for leaks around the mouth and a nose clip should be used while the investigator holds the subjects’ cheeks. Additionally, it is important that a stable period of tidal breathing is achieved. The specific operations are as follows: 1 min of data collection per session, and quality control processes are performed to identify common artefacts such as leaks, swallows, coughs, and incorrect tongue placement. Real-time display of volume, flow and pressure traces allows the operator to identify the presence of artefacts, which in most situations requires repeating acquisitions until at least three measurements have been recorded that are free of artefacts.

The following parameters were recorded: total respiratory impedance (Zrs), resonant frequency (Fres), respiratory system resistance (R_5_, R_10_, R_15_, R_20_, R_25_, R_35_, collectively referred to as Rrs), fall in resistance between R_5_ and R_20_ (R_5_–R_20_Hz), and respiratory system reactance (X_5_, X_10_, X_15_, X_20_, X_25_, X_35_, collectively referred to as Xrs) at oscillation frequencies of 5, 10, 15, 20, 25, and 35 Hz.

### Statistical analysis

Descriptive statistical methods (median, mean ± SD and frequency) were used to analyse the demographic data and the parameters of IOS in different age stages. Analysis of differences between groups was performed with the Mann–Whitney test. Correlations between respiratory parameters and weight or height were analysed with Pearson’s correlation. Stepwise multiple linear regression was performed to identify the best predictor among various parameters of the IOS by using height, age, body weight, and sex as potential independent variables.

Pearson’s correlation was used to compare the correlation between the present reference equation and other reference equations. All statistical analyses were performed using IBM SPSS Statistics version 20 (IBM, Armonk, NY, USA). P < 0.05 was considered statistically significant. Regression lines were generated using the linear regression methods by GraphPad Prism 9.

## Results

### Demographic features of the participants

**Fig. 1:**
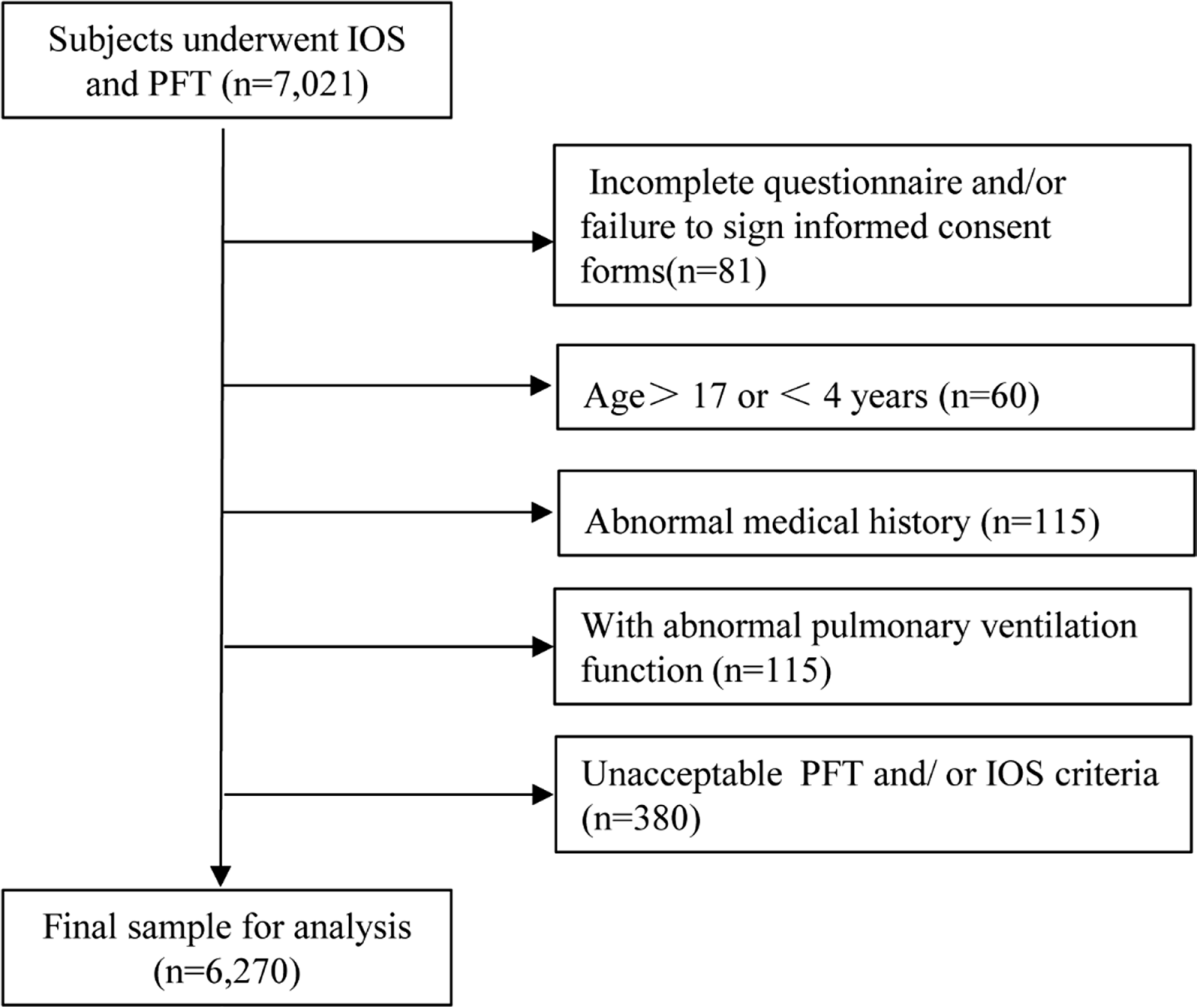
Flowchart of subjects’ exclusion after quality check. *ATS* American Thoracic Society, *ERS* European Respiratory Society

A total of 7021 children were measured with IOS and PFT. The reasons for exclusion were (1) incomplete questionnaire and/or failure to sign informed consent forms (n = 81), (2) failure to fall within the predetermined age range (n = 60), (3) having health problems according to the questionnaire (n = 115), (4) inability to complete IOS and/or PFT (n = 380), and (5) abnormal pulmonary ventilation function (n = 115). Finally, 6270 healthy children (3345 males, 2925 females) were included in the statistical analysis (Fig. 1), with a mean age of 10.36 ± 3.56 years (range: 4–17.0 years; interquartile range: 7.64–13.00 years), a mean height of 144.36 ± 18.96 cm (range: 67–186 cm; interquartile range: 130–160 cm), and a mean weight of 40.41 ± 16.86 kg (range: 14–89.00 kg; interquartile range: 26.5–51.68 kg) (Table 1).

**Table 1:** Summarizes the demographic data of the participants

Age, years	Boys			Girls		
	N	Height, cm	Weight, kg	N	Height, cm	Weight, kg
4	62	109.97 ± 7.51	19.56 ± 4.39	25	109.28 ± 4.84	17.91 ± 1.75
5	168	115.16 ± 6.12	21.82 ± 4.72	132	114.91 ± 5.69	20.01 ± 2.90
6	322	122.29 ± 6.72	24.22 ± 5.09	265	121.51 ± 6.25	21.93 ± 3.20
7	301	128.46 ± 6.58	27.68 ± 5.61	248	127.26 ± 5.84	25.07 ± 3.53
8	326	132.98 ± 7.05	30.78 ± 7.49	301	131.95 ± 6.50	27.88 ± 4.69
9	331	139.33 ± 7.39	35.89 ± 9.85	290	137.21 ± 7.33	31.10 ± 5.97
10	309	144.88 ± 7.13	440.58 ± 11.57	295	144.35 ± 7.94	36.04 ± 7.32
11	312	149.65 ± 7.68	43.85 ± 10.60	254	150.35 ± 7.38	40.23 ± 7.59
12	238	157.13 ± 10.14	50.19 ± 15.30	230	155.77 ± 6.78	45.50 ± 8.05
13	265	163.99 ± 8.56	54.90 ± 13.60	241	159.30 ± 6.58	50.20 ± 7.83
14	193	168.30 ± 7.87	59.87 ± 15.53	185	160.06 ± 7.10	51.64 ± 8.59
15	195	172.05 ± 7.89	64.29 ± 14.03	178	161.50 ± 5.88	53.54 ± 7.83
16	169	174.49 ± 6.26	66.32 ± 13.25	141	162.42 ± 6.52	55.57 ± 8.95
17	154	174.32 ± 6.03	68.83 ± 15.47	140	161.69 ± 5.67	55.07 ± 7.97
Total	3345			2925		

### Comparison of pulse oscillation parameters in children of different age stages and sexes

First, we compared the IOS parameters in different age stages and sexes. As shown in Table 2, there were significant differences in IOS parameters among different age stages. The values of Zrs, Fres, R_5_, R_20_, and R_5_–R_20_ decreased with increasing age, while the X_5_ values increased. Sex was shown to have different influences on IOS parameters in different age stages. For preschoolers, there was no significant difference between the sexes in the main parameters of IOS. However, the IOS values were significantly different between school-age and teenage students of different genders. For school-age students and teenagers, the Zrs, Fres, R_5_, R_20_, and R_5_–R_20_ values of girls are higher than those of boys, while the X_5_ values are lower (Table 2). Our results are similar to those published previously [20–22].

**Table 2:** Mean for resistance at 5 Hz, resistance at 20 Hz, reactance at 5 Hz, impedance at 5 Hz, and resonant frequency stratified by sex and age

IOS parameters	Boy [M(P_25_–P_75_)]	Girl [M(P_25_–P_75_)]	*Z*	*P*
Preschoolers				
R_5_, kPa/L/s	0.88 (0.66–1.12)	0.83 (0.62–1.09)	− 1.409	0.159
R_20_, kPa/L/s	0.57 (0.41–0.78)	0.52 (0.39–0.71)	− 2.438	0.015
X_5_, kPa/L/s	− 0.29 (− 0.4 to − 0.19)	− 0.29 (− 0.39 to − 0.17)	− 0.682	0.495
Z_5_, kPa/L/s	0.93 (0.70–1.18)	0.885 (0.67–1.16)	− 1.203	0.229
R_5_–R_20_, kPa/L/s	0.27 (0.18–0.38)	0.28 (0.17–0.41)	− 0.229	0.819
Fres, Hz	19.44 (17.57–21.33)	20.03 (17.74–21.94)	− 2.021	0.043
School-ages				
R_5_, kPa/L/s	0.67 (0.49–0.88)	0.62 (0.45–0.84)	− 3.626	0.000
R_20_, kPa/L/s	0.47 (0.34–0.6125)	0.42 (0.32–0.59)	− 3.790	0.000
X_5_, kPa/L/s	− 0.19 (− 0.27 to − 0.13)	− 0.19 (− 0.27 to − 0.13)	− 0.890	0.374
Z_5_, kPa/L/s	0.70 (0.52–0.92)	0.65 (0.48–0.88)	− 3.306	0.001
R_5_–R_20_, kPa/L/s	0.18 (0.11–0.28)	0.18 (0.11–0.27)	− 0.584	0.584
Fres, Hz	17.5 (15.00–19.89)	17.94 (15.19–19.82)	− 2.803	0.005
Teenagers				
R_5_, kPa/L/s	0.28 (0.24–0.35)	0.36 (0.31–0.43)	− 9.395	0.000
R_20_, kPa/L/s	0.23 (0.19–0.29)	0.28 (0.23–0.34)	− 7.505	0.000
X_5_, kPa/L/s	− 0.09 (− 0.11 to − 0.07)	− 0.11 (− 0.14 to − 0.09)	− 8.296	0.000
Z_5_, kPa/L/s	0.30 (0.26–0.37)	0.37 (0.32–0.44)	− 9.659	0.000
R_5_–R_20_, kPa/L/s	0.06 (0.03–0.09)	0.08 (0.05–0.12)	− 5.959	0.000
Fres, Hz	13.09 (10.31–15.49)	13.755 (11.08–16.09)	− 2.743	0.006

R_5_ = resistance at 5 Hz, R_20_ = resistance at 20 Hz, X_5_ = reactance at 5 Hz, Fres = resonant frequency, Z_5_ = impedance at 5 Hz, R_5_–R_20_ = fall in resistance between R_5_ and R_20_

### The relationship between IOS parameters and height, weight, and age

**Fig. 2:**
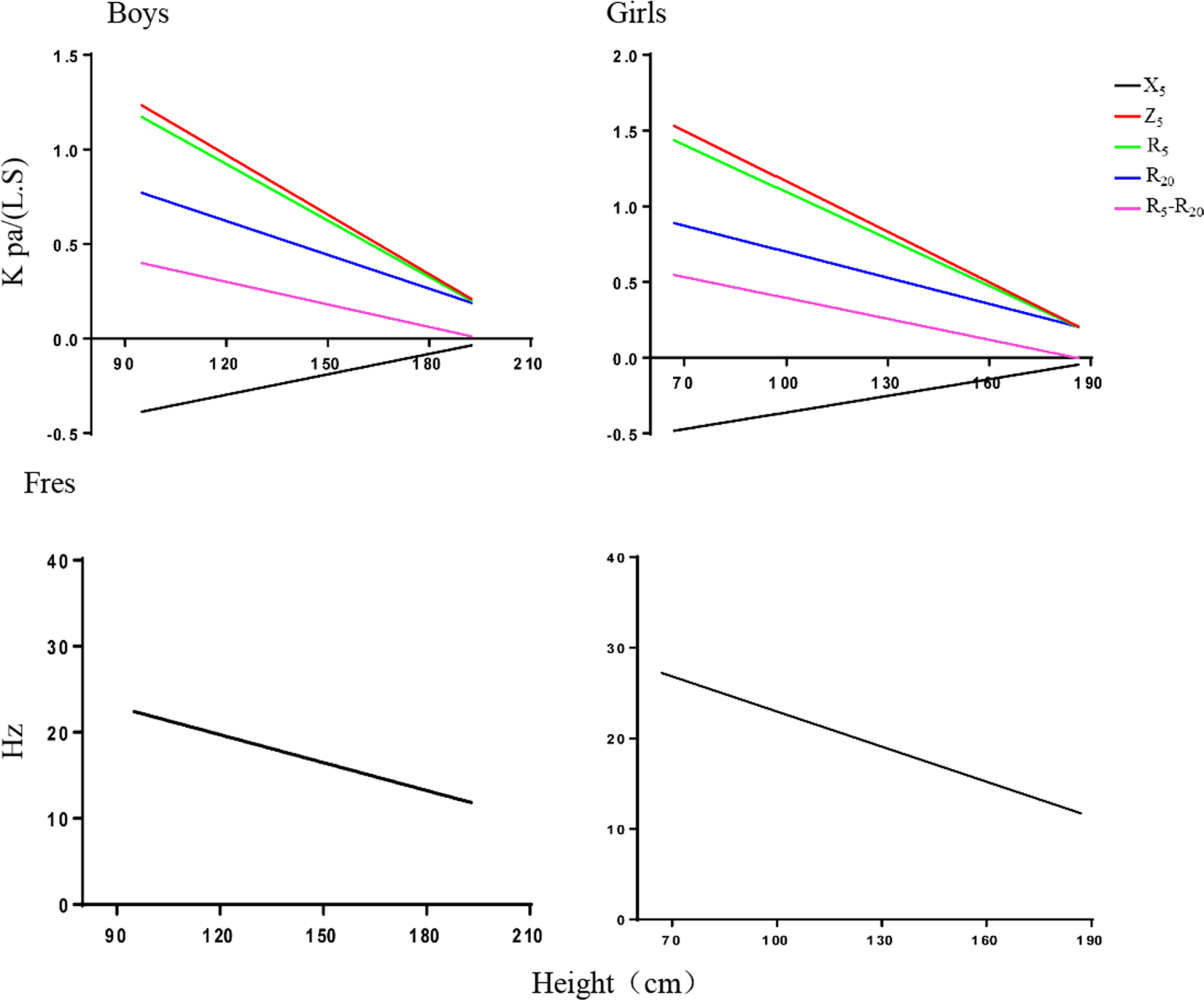
Pearson’s correlation for resistance at 5 Hz, resistance at 20 Hz, reactance at 5 Hz, impedance at 5 Hz, resonant frequency, and reactance curve area below zero for boys and girls. R_5_ = resistance at 5 Hz, R_20_ = resistance at 20 Hz, X_5_ = reactance at 5 Hz, Fres = resonant frequency, Z_5_ = impedance at 5 Hz, R_5_–R_20_ = fall in resistance between R_5_ and R_20_

Next, Pearson’s correlation analysis was applied to examine the relationship between age, height, weight and IOS parameters. We found that IOS parameters were significantly correlated with height, age, and weight. Height was the most closely related to IOS parameters (with the largest R value), followed by age and weight (Table 3). Multiple stepwise regression analysis and further correlation analysis results show that both boy and girl, Z_5_, Fres, R_5_, R_20_, R_5_–R_20_ and are decreased gradually with an increase in height. X_5_ increases gradually with increasing height and is positively correlated with height (Fig. 2; Additional file 1: Fig. S1). Our results showed that height was the best predictor of different IOS outcomes and predicted individual-level psychological outcomes.

**Table 3:** Pearson’s correlation for resistance at 5 Hz, at 20 Hz, at 5 Hz, impedance at 5 Hz, resonant frequency, and reactance curve area below zero for boys and girls

IOS Parameters	Age		Height		Weight	
	r	*p*	r	*p*	r	*p*
Boys						
R_5_, kPa/L/s	− 0.671	< 0.001	− 0.689	< 0.001	− 0.572	< 0.001
R_20_, kPa/L/s	− 0.585	< 0.001	− 0.602	< 0.001	− 0.506	< 0.001
X_5_, kPa/L/s	0.547	< 0.001	0.558	< 0.001	0.465	< 0.001
Z_5_, kPa/L/s	− 0.685	< 0.001	− 0.707	< 0.001	− 0.583	< 0.001
Fres, Hz	− 0.335	< 0.001	− 0.352	< 0.001	− 0.305	< 0.001
R_5_–R_20_, kPa/L/s	− 0.522	< 0.001	− 0.517	< 0.001	− 0.422	< 0.001
Girls						
R_5_, kPa/L/s	− 0.637	< 0.001	− 0.664	< 0.001	− 0.591	< 0.001
R_20_, kPa/L/s	− 0.536	< 0.001	− 0.552	< 0.001	− 0.508	< 0.001
X_5_, kPa/L/s	0.516	< 0.001	0.533	< 0.001	0.492	< 0.001
Z_5_, kPa/L/s	− 0.654	< 0.001	− 0.684	< 0.001	− 0.609	< 0.001
Fres, Hz	− 0.397	< 0.001	− 0.430	< 0.001	− 0.376	< 0.001
R_5_–R_20_, kPa/L/s	− 0.505	< 0.001	− 0.511	< 0.001	− 0.419	< 0.001

R_5_ = resistance at 5 Hz, R_20_ = resistance at 20 Hz, X_5_ = reactance at 5 Hz, Fres = resonant frequency, Z_5_ = impedance at 5 Hz, R_5_–R_20_ = fall in resistance between R_5_ and R_20_

### IOS main parameter prediction equation

IOS was used to determine reference values for pulmonary function. The European Lechtenbörger reference equation (unpublished communication) is widely used in China. Due to ethnic and regional differences, the expected value formula is not suitable for Chinese children. Our findings have shown that height, weight, and age affect the IOS results. Therefore, we calculated the prediction formula of normal IOS values by linear regression. We used multivariate linear by multiple stepwise regression analysis, with age (A), height (H), and weight (W) as the independent variables of the equation and R_5_, R_20_, X_5_, Z_5_, Fres, and R_5_–R_20_ as the dependent variables, to examine different IOS-related parameters for each sex (Table 4; Additional file 1: Table S1).

**Table 4:** Respiratory System Reference Equations Obtained by the Impulse Oscillometry System for Boys and Girls

IOS parameters	Equations	R	R^2^	SE of the estimate
Boy				
R_5_, kPa/L/s	1.894 – 0.007 × H-0.028 × A + 0.002 × W	0.620	0.385	0.264
R_20_, kPa/L/s	1.087 – 0.004 × H-0.010 × A + 0.001 × W	0.608	0.370	0.136
X_5_, kPa/L/s	− 0.554 + 0.002 × H + 0.007 × A	0.566	0.321	0.091
Z_5_, kPa/L/s	1.934 – 0.008 × H-0.017 × A + 0.002 × W	0.716	0.513	0.189
Fres, Hz	31.885 – 0.094 × H − 0.257 × A + 0.035 × W	0.532	0.283	3.615
R_5_–R_20_, kPa/L/s	0.703–0.003 × H − 0.013 × A + 0.001 × W	0.536	0.287	0.133
Girl				
R_5_, kPa/L/s	1.834–0.008 × H-0.0190 × A + 0.003 × W	0.677	0.458	0.176
R_20_, kPa/L/s	0.964 – 0.003 × H-0.009 × A	0.562	0.316	0.123
X_5_, kPa/L/s	− 0.563 + 0.002 × H + 0.006 × A	0.542	0.294	0.089
Z_5_, kPa/L/s	1.970–0.009 × H-0.020 × A + 0.003 × W	0.696	0.484	0.180
Fres, Hz	33.941 – 0.104 × H-0.303 × A + 0.040 × W	0.494	0.244	4.027
R_5_–R_20_, kPa/L/s	0.787 – 0.004 × H − 0.012 × A + 0.002 × W	0.533	0.284	0.130

R_5_ = resistance at 5 Hz, R_20_ = resistance at 20 Hz, X_5_ = reactance at 5 Hz, Fres = resonant frequency, Z_5_ = impedance at 5 Hz, R_5_–R_20_ = fall in resistance between R_5_ and R_20_

### IOS observed value compared with the predicted value of the present study or other reference equation

**Fig. 3:**
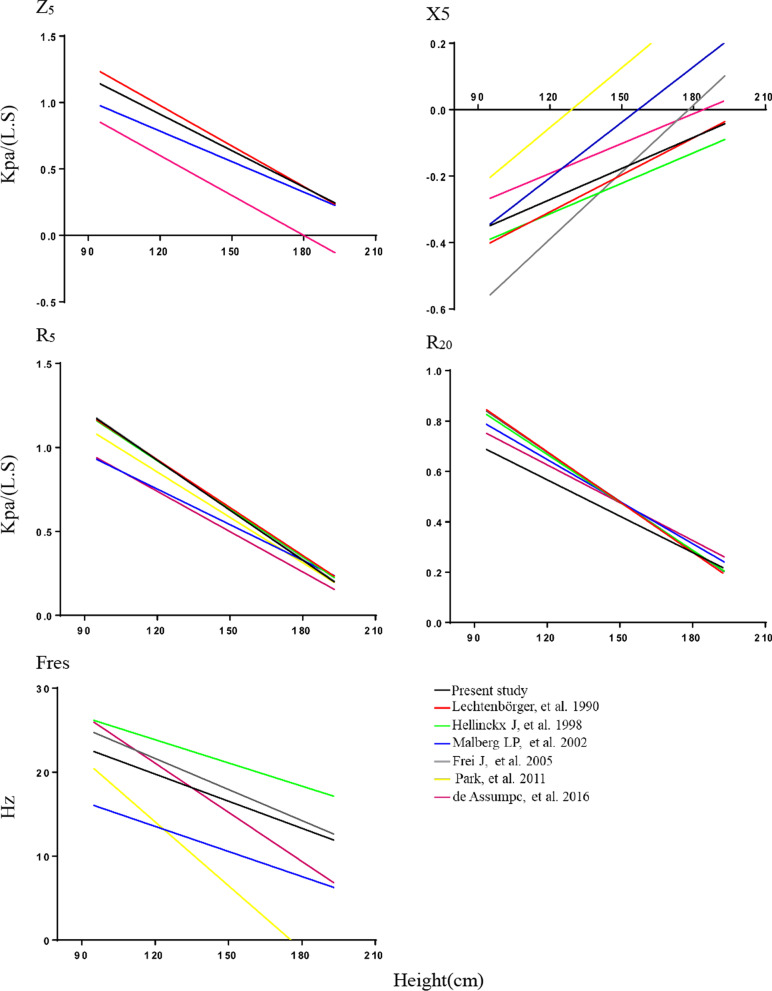
Regression lines obtained in the present study and the other reference equations for boys. R_5_ = resistance at 5 Hz, R_20_ = resistance at 20 Hz, X_5_ = reactance at 5 Hz, Fres = resonant frequency, Z_5_ = impedance at 5 Hz, R_5_–R_20_ = fall in resistance between R_5_ and R_20_

We compared the observed values with the predicted values obtained from the Lechtenbörger reference equation or our reference equation. The obtained correlation coefficients are shown in Table 5. Compared with the Lechtenbörger reference equation, as shown in Table 5, our prediction equation relevance numerical formulas were greater. We also compared the regression lines for boys obtained in the present study and the other reference equations for both sexes. As shown in Fig. 3, there were some differences between the regression lines obtained in the present study and those obtained in other reference equations. The results showed that the consistency (correlation coefficient) of this series of equations for Chinese children was mostly greater than that for previously reported reference equations [8–12] (Table 5; Fig. 3).

**Table 5:** Correlation coefficient between measured values of IOS parameters and predicted values of this prediction equation and lechtenboerger reference equation (r value)

	Lechtenbörger reference equation	The present study equation	Lechtenbörger reference equation	The present study equation
	Boys		Girls	
Zres	0.479	0.643	0.797	0.606
R_5_	0.439	0.622	0.792	0.602
R_20_	0.018	0.568	0.139	0.512
X_5_	0.390	0.516	0.262	0.502
Fres		0.530		0.494

## Discussion

Reference values are essential for interpreting IOS according to the ATS and ERS [17]. However, few studies have developed a prediction equation for IOS parameters worldwide. Most of the existing predictive equations are based on data from white children of European origin [8–14]. Given the differences in race, region, geographical location, and environment, if the interpretation is based on unsuitable reference values, it will cause incorrect results and mislead clinicians. Therefore, it is urgent to establish reference values for IOS parameters among children in China to provide a basis for the rational application of IOS.

Compared with PFT, IOS is a simple, nonspecial cooperative lung function test method that is easy to use and more suitable for elderly, critically ill patients and children who are not easy to cooperate, especially 3–5 young children [23–25]. IOS can measure the resistance and reactance at different frequencies, so it can provide important information on different areas of the lung. According to the frequency dependence and reactance of resistance, it can determine the location and severity of airway obstruction and can be used to monitor the pathophysiological changes of the respiratory system and patients with asthma and chronic obstructive pulmonary disease. It can replace PFT to assist in the diagnosis and follow-up of asthma [26–28].

In total, 6270 healthy children aged 4–17 years old and 100–180 cm in height from 20 regions and 24 research centres in China were enrolled in this research. The results showed that age, height, and weight were closely related to IOS values, among which height was the most important. The second is age, and weight has the weakest effect. With increasing age and height, the total respiratory impedance and airway resistance gradually decreased, while the respiratory system reactance decreased (negative value increased). Recent studies showed that R_5_–R_20_ was a direct measure of anatomical narrowing in the small airways and that small airway narrowing had a marked impact on both asthma control and quality of life [29, 30]. We found that R_5_–R_20_ gradually decreased with increasing age and height, which was similar to airway resistance.

With increasing age and height, the total respiratory impedance and airway resistance decreased gradually, while the negative value of respiratory system reactance decreased (the value increased) and showed the frequency dependence of airway resistance. In healthy children, airway resistance is mainly affected by airway diameter, which is inversely proportional to the fourth power of the airway radius. Due to having a narrow airway diameter, the resistance of young children is greater than that of older children. With increasing age, the diameter widens, and the resistance decreases. In particular, the reduction in peripheral resistance is more significant [31].

Children in the process of growth and development undergo significant increases in their height and weight; in particular, the growth rate of height is ahead of the growth rate of weight. Taking 2-year-old children as an example, according to the WHO standard, the height of 2-year-old children increases by 5–7 cm, and the weight increases by 2 kg every year. The growth of height reflects the growth of bones. With the development of whole-body bones, the thorax increases, and the lung volume, airway diameter and airway surface area increase rapidly. Therefore, the airway resistance of older children is significantly lower than that of younger children. On the other hand, with increasing age, the development of bronchial smooth muscle gradually improves, which increases the elastic retraction force of the lung. Therefore, the lung compliance gradually increases, and the negative value of X_5_ decreases with increasing age. In conclusion, height is the most important factor affecting respiratory impedance, followed by age and weight. The results are similar to those reported by Zheng Jinping [32, 33].

The difference in airway diameter between different sexes is small, especially among young children, so there is no significant difference in the specific parameter value of airway resistance. Many articles show that even in different ways of pulmonary function examination, there are few gender differences in parameters [20–22], but there are significant differences in older children. Our results also found that sex, at different age stages, has different effects on IOS parameters. For preschoolers, gender had no statistically significant influence on the IOS parameters. However, IOS values are different across different genders among school-age children and teenagers; the values of Zrs, Fres, R_5_ and R_20_ were lower among boys than among girls, while X_5_ values were higher among boys. The main reason may be that the height and weight of older children are very different across children of different sexes at the same age; therefore, height and weight—especially height—will significantly affect the results of IOS parameters.

Fres is the oscillation frequency when the reactance is zero, and the respiratory impedance is equal to the respiratory system resistance. It is a sign of the transition from low frequency dominated by respiratory system reactance to high frequency dominated by inertial resistance. It is a sensitive index reflecting the increase in respiratory tract resistance. In children with mild peripheral respiratory tract obstruction, Fres will increase even when R_5_ has no significant change. The fluctuation range is large. It reaches as high as 24 Hz at the age of 4 years and decreases to 14 Hz at the age of 17 years, which tends to be approximately 10 Hz in adults. This indicates that Fres decreases dynamically with age, and this result was similar to the result of Zheng Jinping's [32, 33].

The European Lechtenbörger predictions were widely used in China. The Lechtenbörger prediction equations were based on 614 healthy German children and adolescents from 5 to 17 years in 1990, without excluding smokers and with an uneven number of age and sex groups. The correlation between respiratory function parameters and ethnic differences was obvious, so the reference equations were not suitable for the evaluation of healthy people in China, especially children. The results of this study suggest that height is the most important factor for IOS parameters, followed by age and weight. With increasing age, height and body mass, airway resistance (Rrs) gradually decreases, Fres, which represents lung compliance, and electrical resistance (Xrs) increases gradually. In the present study, the actual measured values are compared with the predicted values of the equations in Tables 4 and 5 and the predicted values of the Lechtenbörger equation. The results show that the correlation coefficient of the series of equations for children in China is significantly greater than that of the Lechtenbörger reference equation. The differences between the two groups of equations and the measured values of respiratory impedance in different height segments are small and similar. However, the differences in the Lechtenbörger reference equation in Zrs, R_5_ and R_20_ are significant. In addition, we also compared the regression lines obtained in this study with other foreign reference equations and found that our prediction equations are different from other reference equations. All these results suggested that foreign reference equations were not suitable for Chinese children.

This study had several limitations. First, similar to other IOS reference studies, this cross-sectional study had limited interpretation of longitudinal changes in lung function. In addition, IOS measurements did not include the area of reactance (AX), which is considered a useful parameter in the evaluation of children with asthma [34, 35]. Finally, the healthy children in this study were defined through the questionnaire survey and did not undergo blood IgE screening and chest X-ray, which could not completely exclude children with allergic diseases and lung disease. Therefore, these issues should be considered in future studies.

## Conclusions

Our research found that age, height, and weight were closely related to IOS values, and height was the most influential predictor variable in most IOS parameters. Our reference equations were developed for the IOS examination in children and adolescents. Compared with other prediction equations, our prediction equations were more suitable for Chinese children and are worth promoting.

## Supplementary Information

**Additional file 1: Fig. S1**. Pearson’s Correlation for Resistance at 5 Hz, Resistance at 20 Hz, Reactance at 5 Hz, Impedance at 5 Hz, Resonant Frequency, and Reactance Curve Area Below Zero for Boys and Girls. **Table S1**. Respiratory System Reference Equations Obtained by the Impulse Oscillometry System for Boys and Girls.

## Data Availability

The datasets analyzed during this present study are available from the corresponding author on reasonable request.
