# Correction: Reference values of impulse oscillometry (IOS) for healthy Chinese children aged 4–17 years

**DOI:** 10.1186/s12931-023-02350-4

**Published:** 2023-02-22

**Authors:** Jinhong Wu, Hao Zhang, Yongsheng Shi, Jinrong Wang, Yuling Han, Qiaoling Zhang, Ning Wang, Sha Liu, Yuehua Zhang, Huifen Zi, Fei Wang, Aihong Liu, Yuxin Song, ChunMei Jia, Yong Feng, Quanhua Liu, liya Wan, Minghong Ji, Zhen Long, Jianfeng Huang, Li Liu, Yun Sun, Suping Tang, Xiaoyan Dong, Xiaojian Zhou, Wenhui Jiang, Li Shen, Haohua Jiang

**Affiliations:** 1 Department of Respiratory Medicine, Shanghai Children’s Medical Center, School of Medicine, Shanghai Jiao Tong University, Shanghai, 200127 China; 2 Department of Internal Medicine, Shanghai Children’s Medical Center, School of Medicine, Shanghai Jiao Tong University, Shanghai, 200127 China; 3 Department of Pediatric Respiratory, Maternity and Child-Care Hospital of Gansu Province, Lanzhou, 730050 China; 4 Department of Pediatric Respiratory, Affiliated Provincial Hospital of Shandong First Medical University, Jinan, 250021 China; 5 Department of Respiratory, Qilu Children’s Hospital of Shandong University, Jinan, 250022 China; 6 Department of Pediatric Respiratory, Maternal and Child Health Hospital in Inner Mongolia Autonomous Region, Hohhot, 010020 China; 7 Asthma Centre of Xi’an Children’s Hospital, Xi’an, 710003 China; 8 Department of Respiratory Medicine, Children’s Hospital of Chongqing Medical University, Chongqing, 400014 China; 9 Pediatrics Infection Disease Ward, Central South University Xiangya School of Medicine Affiliated Haikou Hospital, Haikou, 570208 China; 10 Department of Pediatrics, Baotou Central Hospital, Baotou, 014040 China; 11 Department of Pediatric Respiratory, Guiyang Maternal and Child Health Hospital (Guiyang Children’s Hospital), Guiyang, 550003 China; 12 Department of Respiratory Medicine, Children’s Hospital of Shanxi, Taiyuan, 030013 China; 13 Department of Allergy, Harbin Children’s Hospital, Harbin, 150010 China; 14 Department of Pediatric Respiratory, The Fourth Hospital of Baotou (Baotou Children’s Hospital), Baotou, 014030 China; 15 Department of Pediatrics, Shengjing Hospital of China Medical University, Shenyang, 110004 China; 16 Department of Pulmonology, Xinhua Hospital Affiliated to Shanghai Jiao Tong University School of Medicine, Shanghai, 200092 China; 17 Respiratory Department, Tianjin Children’s Hospital, Tianjin, 300074 China; 18 Department of Pediatric, The First Affiliated Hospital of USTC Anhui Provincial Hospital, Hefei, 230001 China; 19 Department of Pediatric Respiratory Medicine, Maternal and Child Health Hospital of Hubei Province, Tongji Medical College, Huazhong University of Science and Technology, Wuhan, 430070 China; 20 Department of Pulmonology, Children’s Hospital of Fudan University, Shanghai, 201102 China; 21 Department of Pediatric Respiratory, The First Hospital of Jilin University, Changchun, 130021 China; 22 General Pediatric, Yinchuan Women and Children Healthcare Hospital, Yinchuan, 750001 China; 23 Department of Asthma and Tracheitis, Fuzhou Children’s Hospital, Fujian Medical University, Fuzhou, 350000 China; 24 Department of Pulmonology, Shanghai Children’s Hospital, Affiliated to Shanghai Jiao Tong University, Shanghai, 200040 China; 25 Department of Pediatrics, Shanghai First People’s Hospital, Affiliated to Shanghai Jiao Tong University, Shanghai, 200080 China; 26 Department of Respiratory, Guangzhou Women and Children’s Medical Center, Guangzhou, 510623 China; 27 Department of Respiratory, Shanghai Chest Hospital Affiliated to Shanghai Jiaotong University, Shanghai, 200025 China

**Correction: Respiratory Research (2022) 23:182** 10.1186/s12931-022-02080-z

Following publication of the original article [1], the authors identified that the article note about equal contribution was missing.

That is, all authors are contributed equally to the paper. It has been added in this correction and the original article has been updated.

†All authors contributed equally to the paper.
