# Supplementary material for: Reference values of impulse oscillometry (IOS) for healthy Chinese children aged 4–17 years
Source: Respir Res. 2022 Jul 12;23:182. doi: 10.1186/s12931-022-02080-z (PMC9281015; doi:10.1186/s12931-022-02080-z)
Supplement: Supplementary file 1 — Additional file 1: Fig. S1. Pearson’s Correlation for Resistance at 5 Hz, Resistance at 20 Hz, Reactance at 5 Hz, Impedance at 5 Hz, Resonant Frequency, and Reactance Curve Area Below Zero for Boys and Girls. Table S1. Respiratory System Reference Equations Obtained by the Impulse Oscillometry System for Boys and Girls. [file 12931_2022_2080_MOESM1_ESM.docx]

**Figure 1 Pearson’s Correlation for Resistance at 5 Hz, Resistance at 20 Hz, Reactance at 5 Hz, Impedance at 5 Hz, Resonant Frequency, and Reactance Curve Area Below Zero for Boys and Girls**

|  | Boy | Girl |
| --- | --- | --- |
| X_5_ | 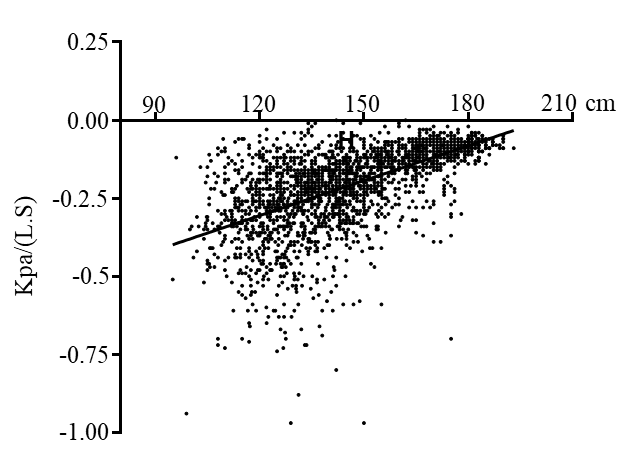 | 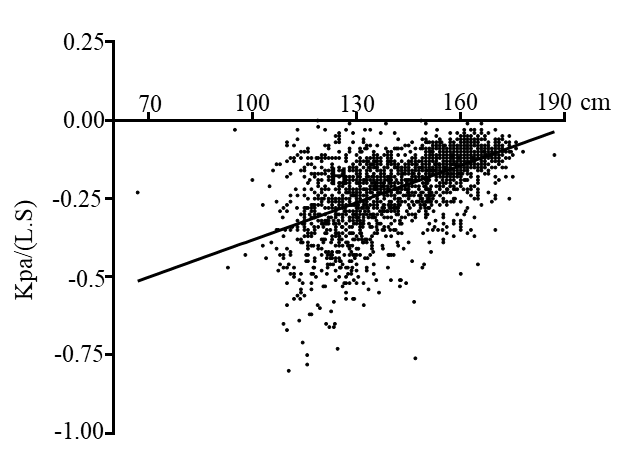 |
| Z_5_ | 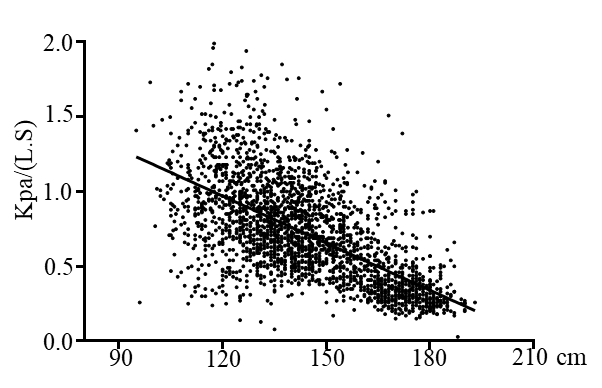 | 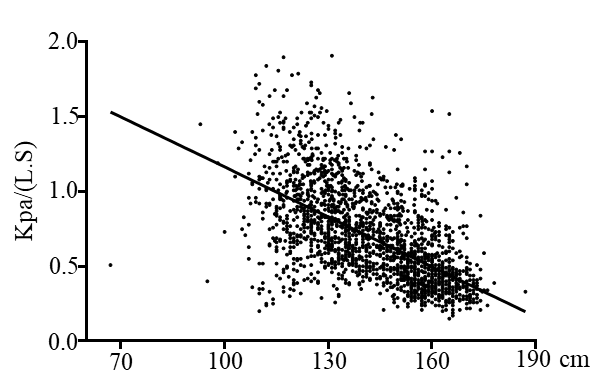 |
| R_5_ | 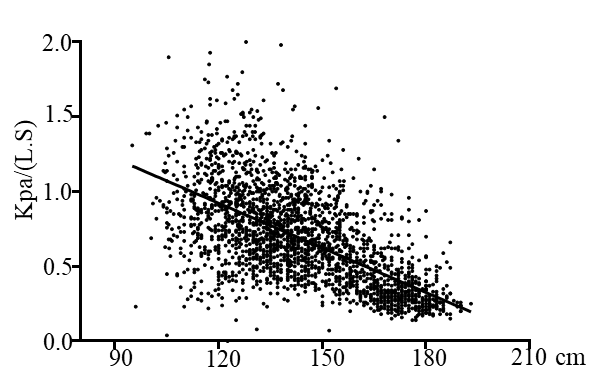 | 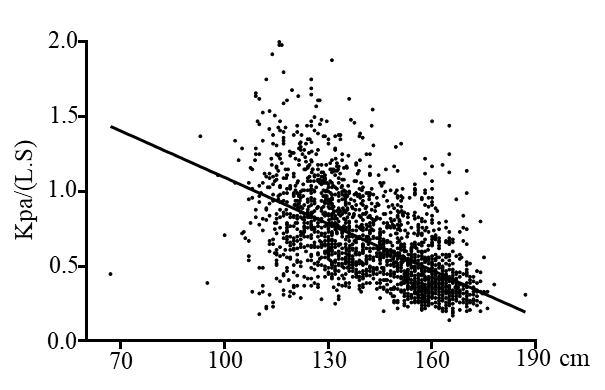 |
| R_20_ | 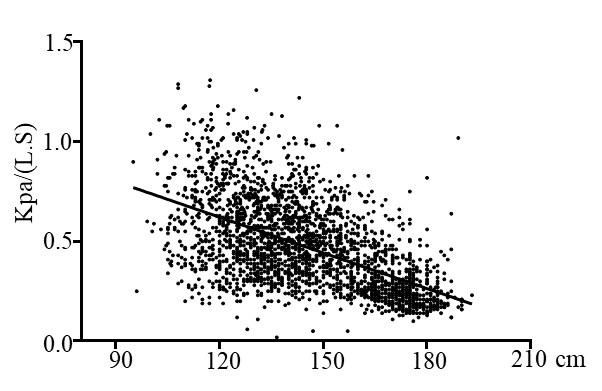 | 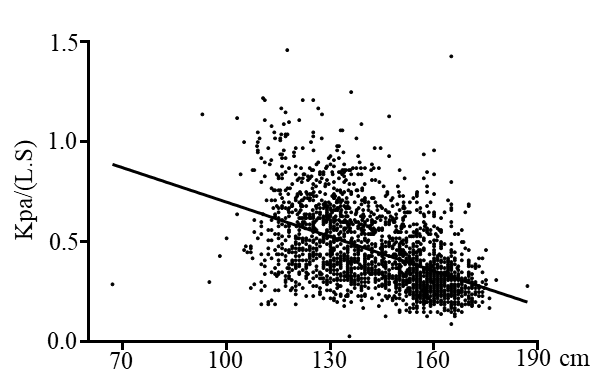 |
| R_5_-R_20_ | 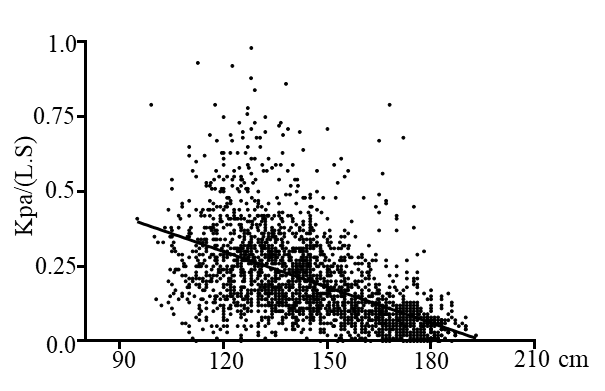 | 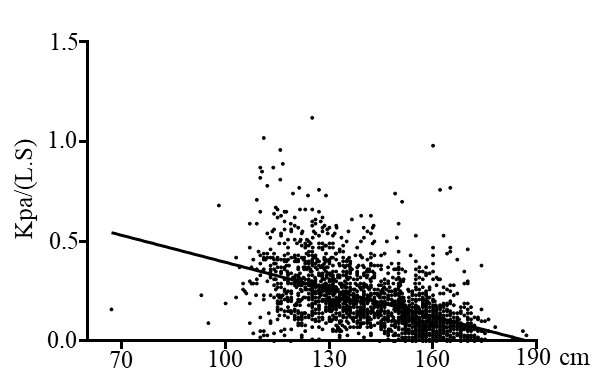 |
| Fres | 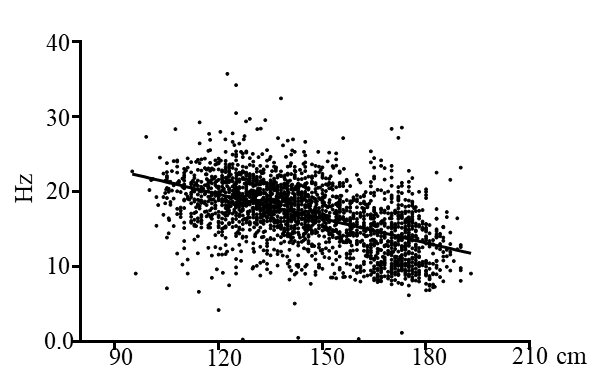 | 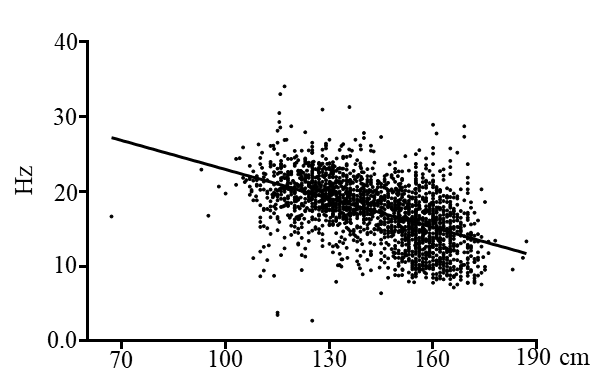 |

**Table 1 Respiratory System Reference Equations Obtained by the Impulse Oscillometry System for Boys and Girls**

| IOS Parameters | Equations | R | R^2^ | SE of the estimate |
| --- | --- | --- | --- | --- |
| Boys | | | | |
| R_10_ | 1.522-0.006×H -0.021×A+0.002×W | 0.629 | 0.396 | 0.201 |
| R_15_ | 1.244-0.004×H -0.016×A | 0.594 | 0.353 | 0.186 |
| R_25_ | 1.124-0.003×H -0.017×A | 0.576 | 0.331 | 0.177 |
| R_35_ | 1.311-0.004×H -0.021×A | 0.593 | 0.351 | 0.200 |
| X_10_ | -0.513+0.003×H +0.008×A-0.001×W | 0.491 | 0.241 | 0.105 |
| X_15_ | -0.390+0.002×H +0.003×A | 0.504 | 0.254 | 0.079 |
| X_20_ | -0.174+0.002×H | 0.304 | 0.093 | 0.075 |
| Girls | | | | |
| R_10_ | 1.346-0.004×H -0.022×A-0.001×W | 0.579 | 0.336 | 0.197 |
| R_15_ | 1.132-0.003×H -0.019×A | 0.543 | 0.295 | 0.183 |
| R_25_ | 0.995-0.002×H-0.019×A | 0.524 | 0.274 | 0.172 |
| R_35_ | 1.173-0.003×H-0.022×A | 0.535 | 0.287 | 0.192 |
| X_10_ | -0.491+0.002×H+0.009×A | 0.524 | 0.275 | 0.090 |
| X_15_ | -0.396+0.002×H+0.006×A | 0.500 | 0.250 | 0.080 |
| X_20_ | -0.256+0.002×H | 0.366 | 0.134 | 0.077 |
